# Perspectives on physician-assisted suicide in mental healthcare: results of a survey of physicians and medical students

**DOI:** 10.1192/bjo.2024.731

**Published:** 2024-08-07

**Authors:** Rebecca Reichel, Sophia Helen Adam, Hans-Jörg Ehni, Florian Junne, Anne Herrmann-Werner, Andreas J. Fallgatter, Stephan Zipfel, Rebecca Erschens

**Affiliations:** Department of Psychiatry and Psychotherapy, University Hospital Tuebingen, University of Tuebingen, Tuebingen, Germany; Department of Psychosomatic Medicine and Psychotherapy, University Hospital Tuebingen, University of Tuebingen, Tuebingen, Germany; Institute of Ethics and History of Medicine, Eberhard Karls University Tuebingen, Tuebingen, Germany; Department of Psychosomatic Medicine and Psychotherapy, Otto von Guericke University Magdeburg, University Hospital Magdeburg, Magdeburg, Germany; and German Center for Mental Health, Magdeburg, Germany; Tuebingen Institute for Medical Education, University of Tuebingen, Tuebingen, Germany; and German Center for Mental Health, Tuebingen, Germany; Department of Psychiatry and Psychotherapy, University Hospital Tuebingen, University of Tuebingen, Tuebingen, Germany; and German Center for Mental Health, Tuebingen, Germany; Department of Psychosomatic Medicine and Psychotherapy, University Hospital Tuebingen, University of Tuebingen, Tuebingen, Germany; and German Center for Mental Health, Tuebingen, Germany

**Keywords:** Depressive disorders, physician-assisted suicide, medical profession, medical survey, ethical perspective

## Abstract

**Background:**

Physician-assisted suicide (PAS) is typically associated with serious physical illnesses that are prevalent in palliative care. However, individuals with mental illnesses may also experience such severity that life becomes intolerable. In February 2020, the previous German law prohibiting PAS was repealed. Patients with severe mental illnesses are increasingly likely to approach physicians with requests for PAS.

**Aims:**

To explore the ethical and moral perspectives of medical students and physicians when making individual decisions regarding PAS.

**Method:**

An anonymised digital survey was conducted among medical students and physicians in Germany. Participants were presented with a case vignette of a chronically depressed patient requesting PAS. Participants decided on PAS provision and assessed theoretical arguments. We employed generalised ordinal regression and qualitative analysis for data interpretation.

**Results:**

A total of *N* = 1478 participants completed the survey. Of these, *n* = 470 (32%) stated that they would refuse the request, whereas *n* = 582 (39%) would probably refuse, *n* = 375 (25%) would probably agree and *n* = 57 (4%) would definitely agree. Patient-centred arguments such as the right to self-determination increased the likelihood of consent. Concerns that PAS for chronically depressed patients might erode trust in the medical profession resulted in a decreased willingness to provide PAS.

**Conclusions:**

Participants displayed relatively low willingness to consider PAS in the case of a chronically depressed patient. This study highlights the substantial influence of theoretical medical-ethical arguments and the broader public discourse, underscoring the necessity of an ethical discussion on PAS for mental illnesses.

Situations may arise in a patient's life that seem so hopeless that death is considered to be the only possible alternative. Resignation can be expressed in a variety of ways, ranging from passive wishes to die to suicide planning or requests for assisted suicide, and it can be present in people with chronic physical illness as well as those with chronic mental illness. According to the definition of the German Ethics Council,^1^ the term ‘assisted suicide’, used hereafter in this paper, refers to the assistance of a third party in the ‘preparation or execution of an autonomous suicide’.

In February 2020, the German Federal Constitutional Court overturned the ban on physician-assisted suicide (PAS) that had been in effect in Germany since 2015 and declared a general ban on assisted suicide to be unlawful. Various legislative drafts for a new regulation have been under intense discussion in the German Parliament but have not found the majority necessary to be enacted.^2^ In Germany, the amendment to the law has led to intensified public debates over the past years, particularly about the role of physicians in suicide assistance.^3,4^ Medical ethicists have stated that a general ethical ban on assisted suicide is untenable for the medical profession, as the underlying fear of a loss of trust in the medical profession cannot be sufficiently substantiated either empirically or ethically. Owing to their qualifications, physicians are considered to be in a position to assess a patient's decision-making capacity and at the same time provide professional suicide assistance.^5^

In their statement *Suicide – Responsibility, Prevention and Self-determination*, the German ethics council aimed to highlight the different responsibilities of various stakeholders in the context of suicide decisions and prevention, explicitly including the medical profession.^6^ The German Society for Psychiatry and Psychotherapy partially follows that line of argument in stating that the examination of free will and the assurance of an informed decision are responsibilities of physicians. In the case of indications of impaired self-determination, it is proposed that expert assessments should be carried out by psychiatric specialists.^7^ Medical ethicists argue that such an evaluation of a patient's decision-making capacity is only ever valid for a specific time and decision, based on the patient's abilities in that moment.^8^ The UN Convention on the Rights of Persons with Disabilities (which include mental health disabilities) emphasises the aspect of autonomy, in that people with disabilities have legal capacity on an equal basis with others.^9^ The Committee on the Rights of Persons with Disabilities further advocates a supported decision-making process that should always rely on an individual's will and preferences regardless of the person's mental capacity.^10^

Following the Federal Constitutional Court ruling in February 2020, representatives of the German medical profession emphasised that physicians are explicitly not obliged to participate in assisted suicide. This was based on the long-held position that ‘the physician's involvement in suicide is not a medical task’.^11^ Although PAS was initially intended for those suffering from physical illness or in palliative care, the international debate has gradually expanded to include people with mental illness as eligible for PAS.^12–14^ Suicide and suicidal ideation form a distinct syndrome that is found in a variety of mental illnesses, with major depression being the most prevalent. Suicidal tendencies are often reported during depressive episodes, with a risk of suicide of around 15%.^15,16^ Owing to the frequent co-occurrence, suicidality is also a diagnostic criterion for severe depression.

In the Netherlands, PAS has been legalised for depressed patients for more than 10 years, with specific precautions designed to ensure that the request is both autonomous and motivated by unbearable suffering.^17^ The reported cases indicate that 1.3% of all instances in 2022 involved patients diagnosed with psychiatric disorders.^18^ Despite the relatively stable nature of this percentage over recent years, the topic has triggered an intense international debate surrounding PAS for individuals with depression. In a similar vein, various surveys have shown that medical professionals in Germany are repeatedly confronted with requests for assisted suicide,^19–21^ many of which come from individuals suffering from chronic depression.^22^ A systematic review by Levene and Parker^23^ further revealed that the majority of requests for PAS among depressed patients were rejected, highlighting the dilemma faced by both physicians and patients. Some of the patients turned instead to Swiss assisted suicide organisations such as Dignitas. In the decade leading up to 2020, an average of 82 German citizens received suicide assistance from Dignitas, with the number dropping to nine patients following the court ruling in 2020.^24^ A frequently cited argument against PAS for depressive patients is that the assessment of a patient's decision-making capacity cannot be conducted with sufficient certainty.^25,26^ However, this assumption is challenged by PAS advocates, who reference common assessment procedures used in decisions related to the rejection of life-prolonging measures, exemplified by advanced decision-making.^27–30^ Emphasising the importance of evaluating patient autonomy on an individual basis, advocates assert that this approach helps prevent discrimination against the mentally ill.^31^ Furthermore, they argue that there is no empirical evidence suggesting that the mentally ill are disproportionately receiving assistance in suicide.^32^ However, the principle of patient's best interests can be invoked in both directions: the protection of a person's life^33^ is weighed against the potential ongoing suffering caused by a chronic mental illness or an unassisted suicide attempt.^34^ Regarding the non-maleficence principle, PAS is seen as contradicting the physician's professional mission of healing patients.^33,35^

Regarding the stance and perspectives of physicians on PAS, concerns commonly revolve around the potential consequences of legalisation. One prevalent fear is the normalisation of PAS, with accompanying apprehensions that physicians and relatives might prematurely give up the fight for life.^33,36^ In addition, the loss of the public's trust in the medical profession is feared.^35^ Conversely, proponents argue that healthcare professionals play a crucial part in safeguarding individuals expressing a wish for PAS from making premature, involuntary or ill-informed decisions, ultimately serving the broader goal of protecting life.^37^ Accordingly, as part of a physician's professional ethos, recognition of a self-determined wish to die is seen as contingent upon detailed, open consultation and careful consideration.^38^

Systematic research on medical professionals’ attitudes on PAS indicates generally low acceptance, reflecting a heterogeneous landscape of reasons. These include considerations of religious beliefs, bioethical arguments and, notably, concerns regarding patient autonomy.^39^ Despite this, the role of healthcare professionals in PAS in the case of chronic mental illness remains relatively underexplored in the existing literature. To address this gap, we conducted an anonymous survey to explore the views of medical students and physicians on PAS for chronically depressed patients. Our objective was to explore the ethical and moral considerations and attitudes relevant to individual decisions about PAS from the perspective of healthcare professionals, the participants’ views and fears about their individual work, the overarching implications for the medical profession as a whole, the physician–patient relationship and the perception of the profession in society.

## Method

### Materials and procedures

A cross-sectional survey was conducted to analyse the views of physicians and medical students on PAS for patients with depression. The questionnaire (see Supplementary material available at https://doi.org/10.1192/bjo.2024.731) was designed by a team of experts based on a literature review of the ethical and sociopolitical debate on the subject. As there is no valid instrument on PAS in chronic mental illness, a 23-item questionnaire with four free-text questions (mostly Likert-scaled) was developed. The piloting of the questionnaire was based on an extensive literature review with students of medical ethics. The questionnaire was further developed deductively by a team of experts during an interdisciplinary summer school.

The survey was sent to several medical and professional associations for general medicine, psychiatry, psychosomatics and palliative medicine, as well as to other platforms for physicians such as Coliquio and the Alliance of Young Doctors. The associations and platforms then forwarded the survey to their respective members. To target medical students, the survey was also sent to the mail addresses of the target groups noted in the online register of the German Medical Association. The target group of students were asked to participate in medical student member groups on Facebook.

The survey was conducted online and anonymously in the period before the decision of the Federal Constitutional Court in early 2020. After an optional assessment of demographic data, participants received a case vignette of a patient with depression and were asked to make a decision in the role of the treating physician. The patient was described as suffering from a severe, chronic depressive disorder that had not gone into remission after numerous therapeutic attempts, including in-patient therapy, medication and electroconvulsive therapy. The patient had been treated by her physician for many years, and her wish to die was described as persistent (see Box 1). Participants were also asked to rate relevant ethical arguments according to their personal importance on a Likert scale.

Box 1 Illustration of the case vignette that was presented to the participants to elicit their views and attitudes.

Mrs Müller, aged 60, has been suffering from a severe, chronic depression since her adolescence and has undergone numerous unsuccessful in-patient and out-patient treatments including psychotherapy, various medications, sleep deprivation and electroconvulsive therapy.

The patient states that she has lost all hope of improvement. Her suffering is unbearable and she can no longer see any meaning to her existence. The patient is unable to cope with daily activities and appears to be in a state of neglect. Mrs Müller is not acutely suicidal and is responsive during conversations. Her verbalised wish to die is chronic and has remained unrelenting over time, including during the course of psychotherapy. Her social life is characterised by loneliness and isolation that is caused by the disease.

Mrs Müller's treating physicians see only a small chance of improvement in her current condition and have classified her depression as resistant to treatment. The patient fulfils the diagnostic criteria for a double depression, as she has been suffering from a persistent state of melancholy (dysthymia) for more than 2 years, in combination with episodes of major depression several times per year. Other similar cases to that of the described patient have shown that affected individuals will continue to meet the diagnostic criteria for chronic depression for several years.

As Mrs Müller's treating physician, you have known her for a long time. Mrs Müller has repeatedly asked you to support her in her intention to die and to provide her with medication for this purpose, as she wishes to be sure not to wake up again and to die without pain.

### Data analysis

Data analysis was performed using Stata/SE 16.1. The associations of personal attitudes with individual willingness to provide suicide assistance in the situation described in the case vignette were examined using generalised ordinal logistic regression. This method assesses the respective influence of independent variables on an ordinally scaled target variable.^40^ In this analysis, the magnitude and direction of the influence of various independent variables on the ordinal outcome variable ‘participants’ willingness to provide suicide assistance’ were determined.

### Development and implementation of the ordinal regression model

First, the requirements for ordinal regression were checked using Stata. Multicollinearity of the independent variables was checked using the Stata command ‘collin’. None of the variables was found to have a variance inflation factor in the borderline range (≥10), so the requirement could be considered to be met. In a second step, the Brant test^41^ was used to test for the further prerequisite of a proportional odds ratio. A significant result of the Brant test was found for some of the independent variables (questions 11.2, 13, 14, 15 and 16), so that the assumption of parallel regression (proportional odds ratio) had to be rejected. According to the literature, violation of this assumption is common and probably due to the exploratory study design.^42^ This led to a group distribution of the dependent variable that corresponded to the free distribution among all respondents. This, in turn, resulted in significant differences in the group sizes of the dependent variable. The result was a generalised ordinal regression in which the assumption of proportional odds ratios could be relaxed for some variables.^40^ Examination of the respective influence of each independent variable was carried out using the ‘margins’ command. This is used to derive average probabilities for the occurrence of a condition. In the case of this work, it was explicitly not intended to make a prediction (e.g. for a 19-year-old medical student) but only to examine the magnitude and direction of each possible influence on decision-making.

### Qualitative analysis with regard to Mayring

After reading the case vignette, participants were asked to decide and then provide a free-text justification for their decision. The free-text justification allowed a qualitative analysis of the arguments used. This was done by coding the free-text responses according to Mayring's content analysis method.^43^ In this analysis, the magnitude and direction of the influence of various independent variables on the ordinal outcome variable ‘participants’ willingness to provide suicide assistance’ was determined.

### Ethics

The responsible Ethics Committee of the University Hospital and Medical Faculty of the University of Tübingen was informed about this work (project number 179/2023A). For completely anonymous data, consultation with and approval by the ethics committee regarding data collection, analysis and publication are not required. Written informed consent for participation was not required for this study, in accordance with national legislation and institutional requirements. It should be emphasised that this analysis was performed within a descriptive framework and in no way reflects the opinions or initiatives of the authors.

## Results

### Demographics

The participants included in the survey were 32% (*n* = 478) male and 61% (*n* = 911) female; 6% (*n* = 95) did not specify. The age distribution ranged from *R* = 17–77 years with an average age of 29 years (s.d. = 13). Almost 80% (*n* = 1186) were still studying medicine, whereas 21% (*n* = 292) were already practising as physicians. A significant proportion (57%) of the responding physicians had specialised in palliative care. A majority of the medical students 57% (*n* = 676) had clinical work experience in the healthcare system outside their medical training, and 42% (*n* = 498) had been taught medical ethics as part of their curriculum by the time of their participation.

### Answering the main question

**Fig. 1:**
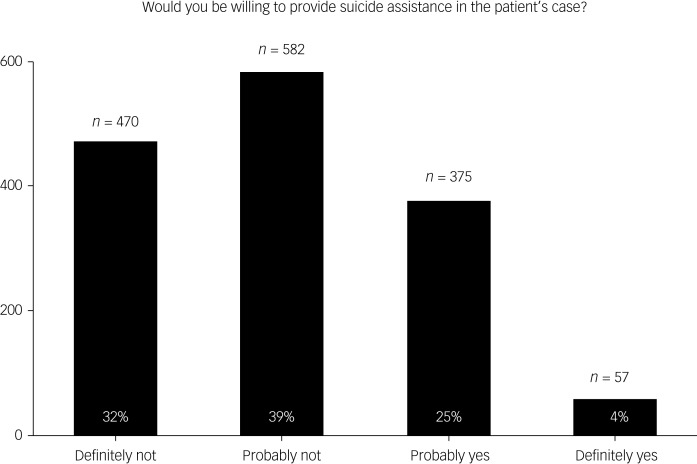
Frequency of agreement with the question ‘Would you be willing to provide suicide assistance in the patient's case?’ among physicians and medical students. The y-axis shows frequency of agreement among participants; the x-axis shows the four possible answer options ‘Definitely not’, ‘Probably not’, ‘Probably yes’ and ‘Definitely yes’.

**Fig. 2:**
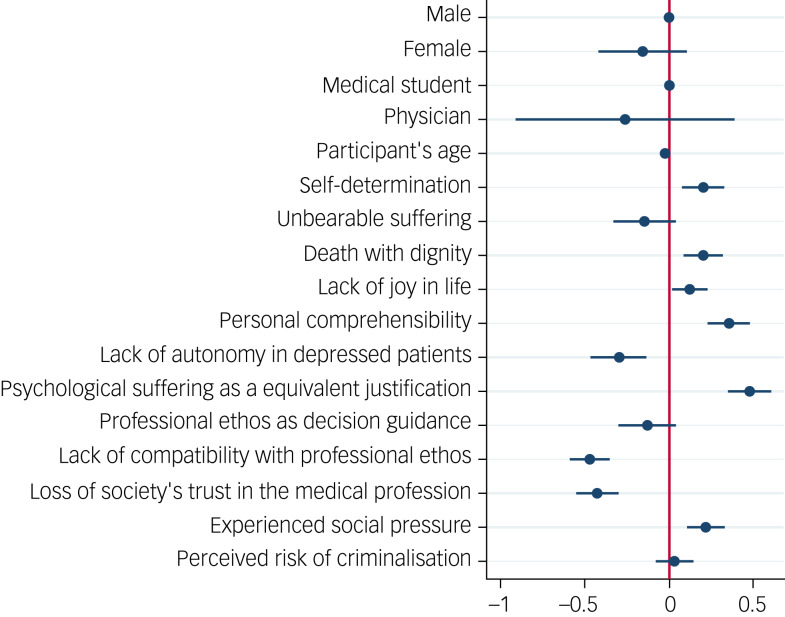
Odds ratios of providing suicide assistance in the case vignette. The x-axis shows summarised odds ratios for each item plotted on the y-axis. The red line indicates zero on the x-axis. A negative coefficient indicates a decreased probability of agreement; a positive coefficient indicates an increased likelihood of agreement.

According to the survey, 71% (*n =* 1052) of participants were not willing to provide PAS in the patient case presented. Of the almost 30% (*n* = 432) who could imagine doing so in principle, only 15% (*n =* 57) were sure of their decision (Fig. 1). The factors influencing openness to providing PAS were calculated using logistic regression, with the number of cases included in the logistic regression (number of observations) being *n =* 1368. The regression model as a whole was found to be significant (χ^2^(23) = 1176.19, *P* < 0.00) with a pseudo *R*² (McFadden) of 0.36. As shown in Fig. 2, individual factors had only a partial influence on the decision for or against PAS. Regarding the demographic factors of gender, age and profession, differences in willingness to provide PAS were only marginal. Evaluation of the various arguments mentioned in the theoretical discussion showed that most had a significant influence on the decisions of participants (Fig. 2).

- Of the possible reasons given for assisted suicide, the arguments regarding self-determination (*β =* 0.20, *P* < 0.001), death with dignity (*β =* 0.2, *P* < 0.001) and lack of joy in life (*β =* 0.12, *P =* 0.02) had significant effects on willingness to provide PAS. Participants who found these arguments more understandable were in general more likely to consent to the patient's request.
- Moreover, unbearable suffering as a reason for PAS had a positive influence on the likelihood of providing PAS, although this was not significant for all ordinal group comparisons (coef1 −0.15, *P =* 0.11; coef2 −0.02, *P =* 0.80; coef3 0.79, *P =* 0.01). Participants indicating personal comprehensibility as a factor relevant to their individual decision were significantly more likely to provide suicide assistance (coef1 0.35, *P =* 0.00, coef2 0.18, *P =* 0.01; coef3 −0.10, *P =* 0.45).
- The attitude that depressed patients should not be granted access to suicide assistance owing to a lack of autonomy led to a significantly higher probability of refusing suicide assistance (coef1 −0.3, *P* = 0.00; coef2 −0.79, *P =* 0.00; coef3 −0.59, *P =* 0.002). Moreover, less approval of psychological suffering, compared with physical suffering, as an equivalent reason for PAS led to a higher probability of refusing suicide assistance (*β =* 0.48, *P =* 0.000).
- Participants who stated that their decision was guided by their interpretation of a physician's professional ethos were significantly less likely to provide assisted suicide (coef1 0.13, *P =* 0.124; coef2 −0.49, *P =* 0.00; coef3 −0.46, *P =* 0.002). In turn, those who saw no compatibility of assisted suicide with a physician's professional ethos were significantly more likely to reject PAS (*β* = −0.47, *P =* 0.000).
- Concerns about loss of society's trust in the medical profession made participants significantly more likely to reject PAS (*β =* −0.43, *P =* 0.000).
- The feeling of being under social pressure made participants more likely to agree to PAS (*β =* 0.22, *P =* 0.000).
- Concerns about the criminalisation of the medical profession owing to the legal ban on assisted suicide at the time of the survey had no significant influence on willingness to provide suicide assistance (*β =* 0.031, *P =* 0.583).

### Qualitative analysis

**Fig. 3:**
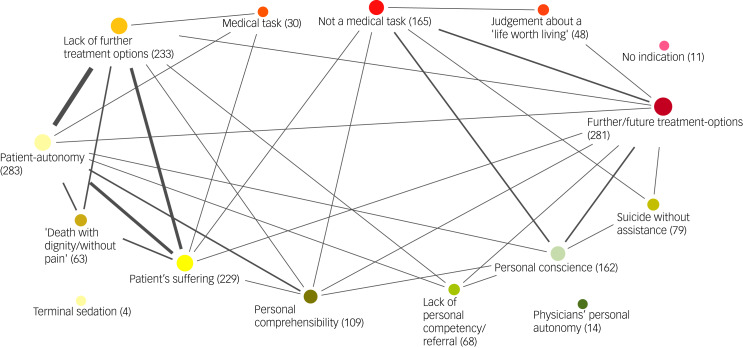
Results of Mayrings's content analysis of free-text responses of all respondents. The size of the dots corresponds to the absolute frequency of the arguments. The thickness of the connecting lines denotes the relative frequency of the links. The patient-focused view (yellow), the physician's view (red) and the personal view (green) are visualised using different colours.

The free-text responses of all the participants painted a heterogeneous picture (Fig. 3). Overall, a patient-focused view, a physician-focused view and a personal view were identified. In the analysis, patient-focused arguments mentioned by respondents could essentially be traced back to the medical-ethical principles of respect for patient autonomy, patient well-being and the avoidance of harm.

From a patient-focused point of view, respondents justified their decision mainly with aspects reflecting the patient's concerns: the arguments regarding patient suffering, patient autonomy and lack of further treatment options were combined. From the physician-focused point of view, respondents referred mainly to their therapeutic mandate and to the protection of life, reflected in the statement that assisted suicide is not a medical task. In addition, they often mentioned further therapeutic options and proposed a wide range of possible interventions for the patient, including aiming to reduce the patient's social isolation (for instance, through support groups and permanent assisted living), as well as medical treatments such as ketamine or deep brain stimulation. In addition, it was pointed out that in the future new therapy methods might be developed.

In the personal view, the justification for one's own decision focuses on various aspects that are not primarily related to the role of the physician or the situation of the patient. Rather, the focus is on the respondent's own feelings and values. The basic arguments of the discussion, such as patient autonomy (*n* = 409), prevention of suffering (*n* = 229), protection of life (*n* = 105) and social justice – mainly in the sense of the ‘slippery slope’ (*n* = 38) – were mentioned by the participants as relevant to their personal decision for or against PAS.

Furthermore, several other clusters of arguments were mentioned. Participants often justified their decision by referring to their own conscience or to a categorical rejection of the request (*n* = 162), without further disclosing the underlying values associated with their decision. Given that the patient had no physical limitations, participants repeatedly stated that the patient was in principle capable of attempting suicide without assistance (*n* = 79).

Another aspect was the participants’ own religious beliefs. In addition, respondents referred to the law, which they said made any personal decision either unnecessary or inadmissible.

Participants also referred to a lack of personal competence to decide on the admissibility of PAS and to carry out the process properly (*n* = 68). They also repeatedly equated PAS with euthanasia and even active killing.

**Fig. 4:**
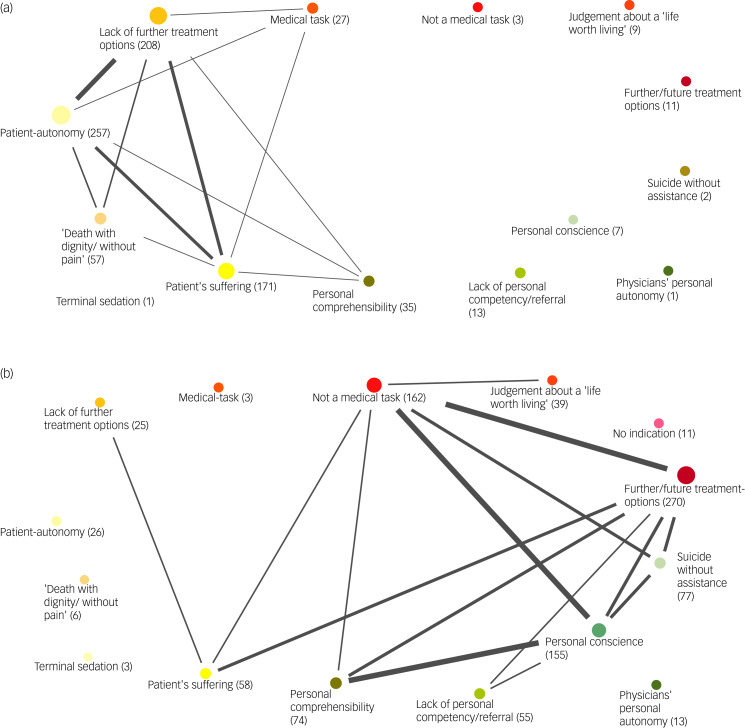
(a) Code map illustrating types and frequencies of reasoning for participants who agreed to physician-assisted suicide (PAS). (b) Code map illustrating types and frequencies of reasoning for participants who opposed PAS.

In a further step, we examined whether people who expressed a willingness to provide PAS (Fig. 4(a)) took a different argumentative stance in the free-text justification than people who rejected PAS (Fig. 4(b)). Among respondents who approved of providing PAS, an increased adoption of the patient-centred view was observed. According to our qualitative analysis of the free-text responses, respondents mostly justified this on the basis of their empathy with those affected. A strong association was found with the argument that the patient was ‘out of treatment options’ (*n* = 288), i.e. that no improvement could be expected from further treatment. Further statistical analysis showed that participants who found the patient-centred arguments of self-determination, dignified death and lack of pleasure in life more understandable were more likely to agree to assisted suicide in the patient's case.

By contrast, for participants who refused assisted suicide in the case vignette, the code map showed an increased acceptance of the medical and personal perspectives.

## Discussion

The aim of this study was to investigate the perspectives of physicians and medical students concerning PAS in the case of a chronically depressed patient. This focus was motivated by the relative scarcity of research on chronic mental illness within the PAS literature.

### Factors influencing attitudes towards PAS

The majority of respondents had already been confronted with a request for PAS in the course of their work. This rather high rate was presumably because a significant proportion (57%) of the respondents had received palliative care training. This aligns with existing research emphasising that requests for PAS are highly relevant in practice.^19–21^ In their individual decision on the presented patient case, a decisive majority of respondents rejected the depressed patient's request for assisted suicide. This stance is consistent with earlier research reflecting a prevailing tendency towards opposition to PAS.^39,44^

Although demographic factors did not distinctly influence survey responses, important arguments in bioethical and public discourse had a pivotal role. Participants’ attitudes towards patient autonomy and patient suffering had a significant influence on their decision regarding the provision or refusal of PAS, as did their professional ethics and possible consequences for the physician–patient relationship, as well as perceived societal pressure. In our exploratory analysis, different lines of argumentation emerged within the free-text responses, reflecting varied emphases and weights of arguments among both proponents and opponents of assisted suicide for depressed patients.

The results of the regression model indicated that the patient's unbearable suffering did not exert a significant influence on the willingness to provide PAS, although in the survey a majority of 65% thought that psychological suffering could serve as an equivalent justification for a wish to die (compared with physical suffering). A plausible interpretation lies in the theoretical discourse, where the argument of reduced suffering is used dually: as a rationale for assisted suicide and as a justification for the necessity of additional therapeutic intervention.^33,34^

### PAS as a patient-focused decision

The significance of patient autonomy emerged prominently among participants, with notable disparities between proponents and opponents of PAS regarding the capability of chronically depressed patients to autonomously decide about PAS. Those favouring PAS were more inclined to consider patient autonomy as an argument. On the other hand, 47% of participants agreed with the statement that depressed patients generally lack the autonomy to decide on PAS. This viewpoint was notably associated with a significantly lower willingness to provide PAS. This aspect seems especially problematic considering the UN Committees stance on the Right of Persons with Disabilities: to avoid discrimination of mental health disabilities, a generalisation for specific groups is never acceptable; instead, a supported decision-making process should always take place on an individual basis and irrespective of a patient's capacity.^10^

There was little evidence in the participants’ responses of an explicit and differentiated assessment of the individual patient's autonomy, considering whether the patient's decision is reflecting their actual will, defined as the ‘manifestation of a person's deeply held, reasonably stable and coherent personal beliefs, values, commitments and conception of the good’.^45^ Given the current scientific concept of decision-making capacity,^8^ which calls for an individual assessment rather than a generalised exclusion of groups, such differentiation would be imperative for a well-considered ethical decision.

### PAS as a personal decision

Another domain identified by the qualitative content analysis comprised personal concerns. Respondents who declined PAS cited reasons related to their own conscience, autonomy and the potential burden of participating in PAS. Personal comprehensibility was mentioned more frequently by the opponents (Fig. 4(a) and (b)), in the sense of being insufficiently qualified. Consequently, self-directed suicide without any assistance was suggested as a possible alternative. However, personal comprehensibility plays an important part for both supporters and opponents of assisted suicide. This seems contrary to the findings of the quantitative analysis, where a greater importance of personal comprehensibility was associated with a significantly higher probability of agreeing with PAS.

The problematic nature of such a perspective is illustrated by Lund et al,^46^ who, using an example, showed that respondents considered suicidality in a person to be more acceptable if the person described suffered from a physical disability or if the respondents themselves suffered from depressive symptoms. This reasoning was also found among the participants: ‘I have already had depressive episodes myself and can therefore understand the patient's incredible suffering’. Similarly, multiple studies from the Netherlands have shown that personal beliefs of medical professionals influence their decisions in ethical dilemmas.^8,46^ As a result of the influence of personal comprehensibility shown in the survey, a more in-depth discussion of this aspect from a medical-ethical perspective would be an important step. Moreover, it might be important to explore to what extent participants’ responses are contingent on their capacity to reflect on PAS within the framework of their professional roles.

### Assisted suicide as a question of professional ethics

The qualitative analysis results reveal that respondents who rejected PAS in the case vignette were significantly more likely to employ arguments that reflected the physician's perspective. In addition, in the free-text responses, it was often stated that PAS should not be considered a medical task, consistent with the enduring public stance of the German Medical Association.^11^ However, from a bioethical standpoint, it is notable that participants provided limited elaboration on the underlying reasoning in their responses, so that the recurring reference to the limited medical responsibility is most likely to be interpreted as a general rejection. In some instances, there was explicit mention of the contradiction between assisted suicide and the medical mission to heal. Participants referred to their therapeutic mandate and their duty to protect life. One example of a personal interpretation can be found in the open feedback section of the survey, in which one participant states:

‘*Medical professional ethics need to be better defined, because I think there is a theoretical one (always let people live/keep them alive as much as possible) and a practical one (if the patient is so bad that they would be better off dead, they should be allowed to die – e.g. palliative)’*.

The qualitative analysis of the survey also uncovered a considerable influence of the interpretation of the medical professional ethos with regard to the physician's perspective: physicians who deemed PAS to be incompatible with the medical ethos were more likely to reject assisted suicide with certainty in the case vignette. It should be noted, however, that this does not provide insight into the individual participant's interpretation of the medical ethos.

### Limitations

Some limitations should be considered when interpreting the results of this study. The issue of PAS in patients with depression is highly complex and there is no universal solution. To the best of the authors’ knowledge, there are no systematic surveys available that provide a sufficiently clear basis for ‘empirical ethics’. A digital survey can probably only offer approximate insight into the thought processes of the respondents. In addition, due to the nature of the study and the time constraints, it was not possible to ask follow-up questions about the interpretation of the answers when analysing the free-text responses. Moreover, the demographic data collected in the survey could not be verified, so the accuracy of the information cannot be guaranteed. The survey was also answered by a very heterogeneous group, particularly in terms of the participants’ level of education.

Owing to the construction of the case vignette and the subsequent required decision, there was a direct confrontation with the topic, which was criticised by some of the participants. The lack of a response option for a palliative concept for the patient was repeatedly mentioned as a possible improvement for the survey. This criticism may have been based on the fact that a large part of the sample consisted of physicians trained in palliative care, which may have led to a possible bias. The ability to give free-text reasons also ensured that all aspects relevant to the respondents could be identified. The analysis of the open-ended responses was carried out as a qualitative approach according to Mayring. This method was chosen as the most appropriate on the basis of the large amount of data and the goal of obtaining a comprehensive overview. The recommended recoding by an independent rater to reduce the subjectivity of the analysis was not carried out, which should be considered when interpreting the analysis.

### Clinical implications

This study provides further insight into the motivations of medical students and physicians regarding their attitudes towards PAS in the case of a chronically depressed patient. The responses highlight the challenges of this scenario, and it can be assumed that assisting a patient suffering from chronic depression to attempt suicide would represent a great emotional burden for most of the respondents in this study. In the qualitative analysis, various domains emerged when (future) physicians were faced with this, including medical and social roles but especially personal motives in the decision-making process. In line with former research on guidelines for PAS in depressed patients,^47^ respondents articulated a great need for guidance in their decision whether to provide PAS or not. This could facilitate a better separation of personal motives without undermining the ethical obligations in their professional role. Further research should explore the decision-making process of health professionals in relation to PAS in chronically depressed patients and identify possible differences in decision-making among people with severe physical illnesses. This would allow other influencing factors, such as religious beliefs or personality traits, to be investigated and help to establish future guidelines.

In summary, this study has demonstrated the high complexity and diversity of the theoretical bioethical discussion on PAS for depressed patients in the medical profession. The analysis of the individual reflections of participants showed the importance of medical ethics training as a foundation for reflection on professional action. International voices are urging the psychiatric community in particular to prepare for the expected challenges of the increasing legalisation of PAS by establishing clear clinical standards and providing appropriate medical training.^32^ Such preparatory training might be able to strengthen individual ethical decision-making, fostering a reflective examination of personal motives and promoting professional ethical behaviour.

## Supporting information

Reichel et al. supplementary material Reichel et al. supplementary material

## Data Availability

The data that support the findings of this study are available on request from the corresponding author, S.H.A. The data are not publicly available owing to data protection requirements. Several results and illustrations are part of a German-language medical doctoral thesis by R.R. In Germany, medical doctoral theses are usually published online; this thesis will be accessible once the article has been published (https://publikationen.uni-tuebingen.de/xmlui/handle/10900/144739).
