# Supplementary material for: Perspectives on physician-assisted suicide in mental healthcare: results of a survey of physicians and medical students
Source: BJPsych Open. 2024 Aug 7;10(5):e141. doi: 10.1192/bjo.2024.731 (PMC11698214; doi:10.1192/bjo.2024.731)
Supplement: Reichel et al. supplementary material [file S2056472424007312sup001.docx]

**Welcome to the Survey** "Physician-Assisted Suicide in Depression"

Please take 15 minutes to answer our questionnaire as spontaneously as possible. Remember, there are no right or wrong answers, and your participation is entirely anonymous. Thank you for your time!

**Question 1:** Please indicate your gender. ☐ Male ☐ Female

**Question 2:** Please indicate your age. Use the slider by clicking and dragging with the mouse. 0-100 years

**Question 3:** Please specify your professional status.

☐ Medical student ☐ Resident physician ☐ Specialist physician ☐ Psychologist

**Question 4:** At which university are you studying medicine? (Dropdown-Menu)

**Question 5:** What semester are you currently in? (Dropdown-Menu)

**Question 6:** In which medical field do you plan to work after graduation? (Dropdown-Menu)

**Question 7:** Do you have practical medical experience outside of your studies? ☐ Yes ☐ No

**Question 8:** Have you been taught medical ethics in your studies? ☐ Yes ☐ No

**Question 9:** Please specify your current medical specialization.

☐ General medicine ☐ Psychiatry ☐ Psychosomatic medicine ☐ Other (please specify)

**Question 10:** In which federal state do you work? (Dropdown-Menu)

**Question 11:** Do you have palliative care training? ☐ Yes ☐ No

**Question 12:** Have you ever been confronted with a request for assisted suicide in your medical practice? ☐ Yes ☐ No

**Please read the following patient case carefully. On the next page, we will ask for your assessment.**

*Patient Case:* *"Mrs. Miller, aged 60, has been suffering from a severe, chronic depression since her adolescence and has undergone numerous unsuccessful inpatient and outpatient treatments including psychotherapy, various medications, sleep deprivation and electroconvulsive therapy.*

*The patient states that she has lost all hope of improvement. Her suffering is unbearable and she can no longer see any meaning to her existence. The patient is unable to cope with daily activities and appears to be in a state of neglect. Mrs. Müller is not acutely suicidal and is responsive during conversations. Her verbalized wish to die is chronic and has remained unrelenting over time, including during the course of psychotherapy. Her social life is characterized by loneliness and isolation that is caused by the disease.*

*Mrs. Müller’s treating physicians see only a small chance of improvement for her current condition and have classified her depression as resistant to treatment. The patient fulfills the diagnostic criteria for a double depression, as she has been suffering from a persistent state of melancholy (Dysthymia) for more than 2 years, in combination with episodes of major depression several times per year. Other similar cases to that of the described patient have shown that affected individuals will continue to meet the diagnostic criteria for chronic depression for several years.*

*As Mrs. Müller’s treating physician, you have known her for a long time. Mrs. Müller has repeatedly asked you to support her in her intention to die and to provide her with medication for this purpose, as she wishes to be sure not to wake up again and to die without pain."*

**Question 13:** Can you imagine complying with the patient's request for assisted suicide?

☐ Definitely yes ☐ Probably yes ☐ Probably no ☐ Definitely no

**Question 14:** Please explain your decision to comply with or reject the request in a few sentences.

**Question 15:** Which aspect(s) of the case influenced your decision? Please check. Multiple answers are possible.

☐ "The patient has already undergone numerous unsuccessful treatment attempts."

☐ "The patient states she suffers to an unbearable extent."

☐ "The patient's wish to die can be considered persistent."

☐ "The patient meets the criteria for a Double Depression."

☐ "You have known the patient as a doctor for a long time."

☐ None of the mentioned aspects

**Question 16:** How do you feel about the following possible justifications for the desire for assisted suicide?

*Self-determination of the patient:*

☐ ... completely understandable

☐ ... rather understandable

☐ ... more or less understandable

☐ ... rather not understandable

☐ ... not understandable at all

*Unbearable suffering:*

☐ ... completely understandable

☐ ... rather understandable

☐ ... more or less understandable

☐ ... rather not understandable

☐ ... not understandable at all

*Dignified dying for the patient:*

☐ ... completely understandable

☐ ... rather understandable

☐ ... more or less understandable

☐ ... rather not understandable

☐ ... not understandable at all

*Lack of joy in life for the patient:*

☐ ... completely understandable

☐ ... rather understandable

☐ ... more or less understandable

☐ ... rather not understandable

☐ ... not understandable at all

**Question 17:** Can you think of other justifications that you can understand? If yes, please list them.

**Question 18:** "I consider it crucial, when considering assisting in suicide in a specific case, whether I can personally understand the patient's wish to die."

☐ Completely agree

☐ Rather agree

☐ Neither agree nor disagree

☐ Rather disagree

☐ Completely disagree

**Question 19:** "Depressive patients should be fundamentally excluded from access to assisted suicide because they cannot make this decision autonomously due to their illness."

☐ Completely agree

☐ Rather agree

☐ Neither agree nor disagree

☐ Rather disagree

☐ Completely disagree

**Question 20:** "The mental suffering of a patient can fundamentally justify a wish for death to the same extent as physical suffering."

☐ Completely agree

☐ Rather agree

☐ Neither agree nor disagree

☐ Rather disagree

☐ Completely disagree

**Question 21:** "In answering questions about physician assisted suicide, I orient myself to the general medical professional ethos."

☐ Completely agree

☐ Rather agree

☐ Neither agree nor disagree

☐ Rather disagree

☐ Completely disagree

**Question 22:** "Physician-assisted suicide is incompatible with the medical ethos."

☐ Completely agree

☐ Rather agree

☐ Neither agree nor disagree

☐ Rather disagree

☐ Completely disagree

**Question 23:** " Physician-assisted suicide in chronically depressed patients by doctors will trigger a loss of trust in society in the medical profession."

☐ Completely agree

☐ Rather agree

☐ Neither agree nor disagree

☐ Rather disagree

☐ Completely disagree

**Question 24:** "I feel pressured in my (future) medical practice by the current situation in Germany by politics/society."

☐ Completely agree

☐ Rather agree

☐ Neither agree nor disagree

☐ Rather disagree

☐ Completely disagree

**Question 25:** How well do you know the current legal regulations regarding physician-assisted suicide?

☐ Very well

☐ Rather well

☐ Neither well nor poorly

☐ Rather poorly

☐ Very poorly

**Question 26:** "Due to the legal regulation on assisted suicide passed in November 2015, there is a risk that physicians will be criminalized." (The regulation states that assisting suicide remains legal unless done for profit and involves a close relative.)

☐ Completely agree

☐ Rather agree

☐ Neither agree nor disagree

☐ Rather disagree

☐ Completely disagree

**Note:** You are on the last page of the questionnaire. Clicking "CONTINUE" will end the survey, and you will not be able to change your answers. Do you have any comments on our survey? We appreciate your feedback.
